# Community disaster exposure and first onset of depression: A panel analysis of nationally representative South African data, 2008–2017

**DOI:** 10.1371/journal.pclm.0000024

**Published:** 2022-04-06

**Authors:** Andrew Tomita, Busisiwe P. Ncama, Yoshan Moodley, Rashieda Davids, Jonathan K. Burns, Tafadzwanashe Mabhaudhi, Albert T. Modi, Rob Slotow

**Affiliations:** 1 Centre for Rural Health, School of Nursing and Public Health, University of KwaZulu-Natal, Durban, South Africa; 2 KwaZulu-Natal Research Innovation and Sequencing Platform (KRISP), College of Health Sciences, University of KwaZulu-Natal, Durban, South Africa; 3 School of Nursing and Public Health, College of Health Sciences, University of KwaZulu-Natal, Durban, South Africa; 4 Africa Health Research Institute, University of KwaZulu-Natal, Durban, South Africa; 5 Department of Global Health, Faculty of Medicine and Health Sciences, Stellenbosch University, Cape Town, South Africa; 6 School of Agricultural, Earth and Environmental Sciences, College of Agriculture, Engineering and Science, University of KwaZulu-Natal, Pietermaritzburg, South Africa; 7 Institute of Health Research, University of Exeter, Exeter, United Kingdom; 8 Department of Psychiatry, School of Clinical Medicine, University of KwaZulu-Natal, Durban, South Africa,; 9 Centre for Transformative Agricultural and Food Systems, School of Agriculture, Earth and Environmental Science, University of KwaZulu-Natal, Pietermaritzburg, South Africa; 10 International Water Management Institute (IWMI-GH)—West Africa Regional Office, Accra, Ghana; 11 School of Life Sciences, University of KwaZulu-Natal, Durban, South Africa; 12 Department of Genetics, Evolution and Environment, University College, London, United Kingdom

## Abstract

Sub-Saharan Africa faces unprecedented disasters, with climate change expected to exacerbate the frequency and severity of unpredictable and stressful catastrophic events. Unlike developed nations, reconstruction in developing nations is hindered by resource constraints, with certain communities potentially experiencing multiple and enduring effects of disasters. Despite the potential danger of such cumulative community disaster exposure on mental health (e.g. depression), large-scale population-level evidence for the region is limited. We investigated the association between exposure to cumulative disaster and the first onset of depression in a nationally representative survey in South Africa. We used panel data from the South African National Income Dynamics Study (SA-NIDS) from 2008–2017, consisting of 17,255 adult study participants who were depression free at baseline. Risk of first depression onset between individuals exposed and unexposed to community disaster was measured, accounting for multiple disaster exposure over time by fitting generalized estimating equation (GEE) regression models. Data on the geographic location of disasters were obtained from the South African government gazette, and mapped with the government delineated SA-NIDS households’ locations. Of the sampled individuals, 2,986 were exposed to disaster during the study duration (17.3%). Increased cumulative community disaster was significantly associated with the likelihood of depression onset (adjusted relative risk [aRR] = 1.20, p<0.01, 95% CI: 1.09–1.33), even after controlling for socio-demographic factors. In sub-group analyses, greater likelihood of depression onset was found among females [but not in men] (aRR = 1.23, p<0.01, 95% CI: 1.09–1.38), Black African [but not in other population group] (aRR = 1.21, p<0.01, 95% CI: 1.09–1.36), lower education attainment group [but not in tertiary and above educational attainment group] (aRR = 1.20, p<0.01, 95% CI: 1.08–1.33), and lower income attainment group [but not in the top income quartile group] (aRR = 1.24, p<0.01, 95% CI: 1.11–1.38), due to cumulative community disaster. Although cumulative community disaster exposure was significantly associated with the first onset of depression, its negative impact may be more pronounced among individuals considered chronically socially vulnerable (i.e. the groups above) in South Africa. Given that many individuals in South Africa rely on social, food parcel relief, and health services from government/public sector, timely access to community-based supportive intervention is needed for disaster survivors, prioritizing socially vulnerable groups to help mitigate problems associated with mental health challenges.

## Introduction

Sub-Saharan Africa has seen an increasing challenge in the occurrence of disasters, these being disruptions of normal conditions that cause great damage by exceeding the local capacity of affected community to mitigate their effect [1]. Although it is widely acknowledged that the occurrence of disasters and their impacts on society may be underestimated in Africa [2], the estimated 1143 natural disasters that occurred between 2000–2019 are believed to be responsible for 46,078 deaths, according to the Centre for the Research on Epidemiology of Disaster’s Emergency Events Database (EM-DAT) [3]. In 2019 alone, Africa’s share of death due to natural disasters accounted for 23.0% of the global total, a 238% increase compared to the 2009–2018 annualized average (6.8%) [4]. South Africa had the continent’s third-highest (54 events) number of natural disaster events since 2000, with two of the most devastating drought events of the century occurring in 2004 (i.e. 15 million people affected) [3] and 2015/2016 (i.e. 14.3 million people affected) [5]. The field of disaster risk reduction (DRR) research increasingly recognizes that natural disasters often have a man-made component. Although we prefer not to differentiate between natural and man-made disasters [6] (e.g. industrial accidents, mass unrest, war), EM-DAT estimates that Africa experienced 1,690 man-made disaster events and 54,755 deaths during 2000–2019, with South Africa being the 10^th^ highest globally in the number of man-made disaster events during this period [7].

As SSA nations are confronted by considerable environmental changes [8–12], a number of systematic reviews/meta-analyses have indicated that disasters [13–17] are associated with psychological challenges in the people affected by such events, with certain groups (i.e. low-income, older) who lack the resources to cope with consequences of disasters being particularly vulnerable [18, 19]. Disasters, as potentially a large-scale unforeseen and sudden event [18, 20], can be an emotionally overwhelming experience (i.e. feeling stunned and disorientation [21]) that undermine people’s ability to function. Even when disasters are not sudden (as in the case of droughts), ecological distress [22], a form of psychological distress related to present or anticipated ecological change, cannot be ignored. Considerable disaster research points to the associated negative psychological challenges, with most empirical studies focusing on ‘cumulative’ disasters, or the impact of exposure to multiple disasters on post-disaster mental health [23–26]. It is possible that experiencing multiple disasters can undermine a sense of ontological security or produce multiple economic shocks [27] that can lead to the erosion of resilience and confidence in the future, which, in turn, can negatively impact mental health, or make people more susceptible to the effects of subsequent disasters [28].

Mental illness, including stress-related mood disorders, such as depression, is a globally recognized sustainable development challenge (United Nations Sustainable Development Goals [SDG] target 3.4 [29]). The potentially greater number and severity of natural disasters in Africa than elsewhere [SDG targets 13.1] [30] may further aggravate fragile public healthcare systems [31] and diminish the availability of mental health care, including in South Africa. As a leading cause of disability globally [32], depression affects 9.8% of people in South Africa [33], with only 3.9% accessing the inpatient and outpatient mental health care that they need, according to a modelling study [34]. Depression is also a major impediment to development and is understood to have the highest negative impact on labor productivity among all medical conditions [35]. In South Africa, workplace-related economic loss due to depression is estimated to be 4.2% of gross domestic product (GDP) [36]. More importantly, the right to disaster risk reduction and mental health should be placed into the context of human rights, as the South African Constitution of 1996 [37] guarantees a “right to an environment that is not harmful to their health or well-being” (Section 24), requires all spheres of government to secure the well-being of the people (Section 41), and designates the organs of state responsible for managing the potential risk of disasters (Schedule 4).

Despite the abundance of evidence from developed nations, there are no national-level evidence-based studies in SSA, to the best of our knowledge, including in South Africa, on the effect of cumulative community disasters on the first onset of depression. Despite the common mantra that disaster is expected to exacerbate the health challenge of poor and vulnerable populations [38, 39], evidence on the impact of cumulative community disasters remains scarce in South Africa, a nation with one of the highest and most persistent levels of poverty levels and inequality in the world [40]. This knowledge gap was addressed in the current study by establishing an association between exposure to cumulative community disaster and the first onset of depression (i.e. depression incidence) using the South African National Income Dynamics Study (SA-NIDS). This is a unique nationally representative panel database designed to monitor the socio-demographic and health of the South African population over time, which is rarely available in SSA.

## Methods

Panel data from the SA-NIDS wave 1 (year 2008), wave 2 (2010–2011), wave 3 (2012), wave 4 (2014–2015), and wave 5 (2017) were used. The SA-NIDS employs a stratified, two-stage cluster sampling design to reach a nationally representative sample of households. All adults within each chosen household were targeted, with consenting study participants completing the survey, including questionnaires that include socio-demographic and clinical data relevant to this current study. The details of the sampling methodology are reported by SA-NIDS elsewhere [41]. As a panel study, the SA-NIDS tracks individuals over time, with attrition ranging between 14.8% - 21.9%, depending on the wave [41].

To better isolate the effect of cumulative community disaster on the first onset of depression (and reduce the likelihood of reverse causation), our current study constructed an incident cohort based on similar methods utilized in previous studies [42, 43]. The purpose of constructing the incident cohort was to ensure that the study participants were free of depression initially, and to monitor for the first depression onset over time, between individuals exposed and unexposed to disaster. Our study incident cohort consisted of participants who screened negative for depression at first entry, and for whom there was a further depression rating score available in any of subsequent waves. This means we excluded all participants positive for significant depressive symptoms at their first entry into the SA-NIDS. The remaining study participants had to have been assessed for depression risk at least twice to be included in the incident cohort. The data were right censored at the earliest observation at which a participant screened positive for depression risk (or at the last observation if they did not screen for depression).

### Outcomes

The main study outcome was depression, which was obtained from the 10-item abridged version of the Center for Epidemiologic Studies Depression Scale (CES-D) in the SA-NIDS Adult waves 1–5 questionnaires. As a commonly-used psychometric valid/reliable instrument [44, 45], the CES-D captures depression-associated symptoms during the past week based on selfreport. Each of the items in the CES-D is based on four possible responses in a Likert format, from 0 = rarely/none of the time (less than 1 day) to 3 = almost/all of the time (5–7 days). With the level of depression increasing with a higher total CES-D score (observed Cronbach’s α = 0.75), we classified a total score of 10 or greater as a cutoff to represent significant depressive symptoms, this being consistent with a previous study [45].

### Community disaster exposure

**Fig. 1:**
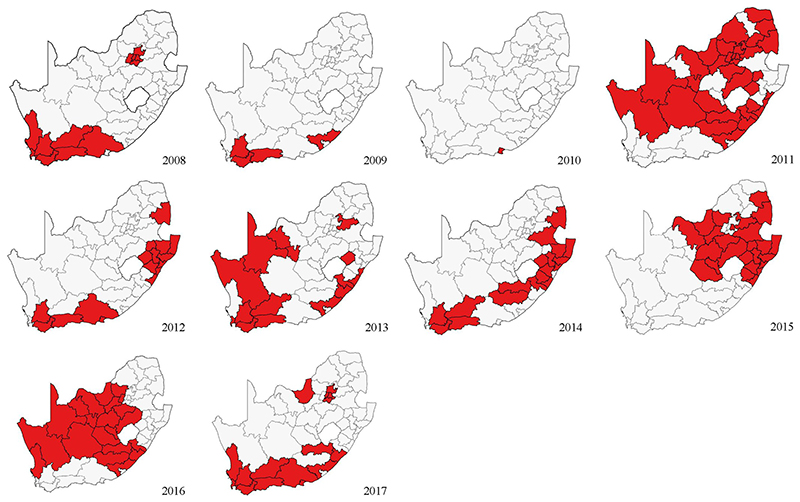
District/metropolitan municipalities impacted by state of disaster declaration according to South African Gazette between 2008-2017. Maps were generated using QGIS 3.10 (https://www.qgis.org/en/site/).

Exposure to a disaster was the study’s main exposure variable, the declaration of the state of disaster and government duties falling under the South African Disaster Management Act, 2002 (Act No. 57 of 2002) [18]. Under sections 1(a) of the Act, disaster is defined as a progressive or sudden, widespread or localized, natural or human-caused occurrence that leads to death or injury, as well as damages to infrastructure or the environment. Sections 1(b) notes that a disaster be of a magnitude that exceeds the ability of those affected by the disaster to cope with its effects including (i) death, injury, or disease, (ii) damage to property, infrastructure or the environment and (iii) disruption of the life of a community. Lastly, a state of disaster declaration, as per sections 27(1), 44(1), and 55(1) of the Act, must be noted in the Government Gazette, the official bulletin that published official or legal notices that contains government agency rules, proposed rules, and proclamations. Data on the state of disaster declaration notices between 2008 and 2017 were obtained from the Gazettes [46], which can be declared nationally, provincially, for any of the nine provinces individually or collectively, or for district or sub-district local municipalities. With a few exceptions, the actual date(s) of the disaster are not indicated in the Gazette, in which case the start date was delineated to be 30 days prior to the Gazette date, due to legislative consultation and publication lag, while the end date was 30 days after the Gazette date. Study participants whose location and interview date fell between the start and end dates (i.e. effective risk period) of the state of disaster were considered to be exposed to the disaster (i.e. reside in a community that was exposed to a disaster) for that specific wave. The lowest geographic unit for publicly accessible SA-NIDS data was district municipality [47], and we regarded a district as being exposed when 50% or more of its local municipalities fell under the state of disaster area. While the Disaster Management Act automatically lapses in three months, the law allows for extensions, with this study extending the period of state of disaster to beyond its three months only for district/local municipalities for scale precision (given large coverage when extended to the entire province). Excluding duplications and extensions, we identified 361 unique location records impacted by the state of disaster declaration between 2008–2017. The map of areas (i.e. district/metropolitan municipalities) impacted by initial state of disaster declaration according to South African Gazette between 2008–2017 are provided in Fig 1. The median length of effective risk period (i.e. the period between the beginning and end of the state of disaster period) was 109 days (IQR: 90–122 days). It is possible that same study participant resided in communities that experienced multiple disaster during the course of study period. Consistent with most disaster research that has tended to study mental health impacts at some stage following events [24, 25], we subsequently constructed a cumulative community disaster exposure variable [hereafter labeled as community disaster exposure] with a running total (i.e. summation of disaster events updated each time a particular community experiences a new disaster event).

### Adverse negative event exposure

Although the primary focus of our study is community cumulative exposure, negative experiences can differ substantially among individuals within affected areas. For this reason, we also utilized negative adverse events measures that are available in the SA-NIDS household questionnaire. The SA-NIDS waves 1–5 captured negative adverse events experienced pertaining to (1) theft, fire or destruction of household property; (2) widespread death and/or disease of livestock, and (3) major crop failure in the last 24 months. Study participants were regarded as being exposed to negative events (based on binary measure) if they reported experiencing any of these three negative adverse events. Consistent with cumulative community disaster exposure, it is also possible that same study participant experienced multiple negative adverse events during the course of the study period. We subsequently constructed a cumulative negative event exposure variable with a running total (i.e. summation of negative events that is updated each time a particular a participant experiences new negative adverse event).

### Covariates

In addition to the socio-demographic data, namely: gender, race, age, educational attainment, residence [urban versus rural], and household income, we used the religious involvement measure available from SA-NIDS Adult questionnaire. Despite the potential benefit of religion, its importance during disasters is often overlooked [48]. In the SA-NIDS, study participant rated the importance of religious activity in their lives by selecting one response in a Likert format: 1 = not important at all; 2 = unimportant; 3 = important; 4 = very important. Subsequently, we created a binary variable of religious involvement where 0 = not important at all/unimportant; 1 = important/very important for our study.

### Ethics statement

Human Research Ethics Committee at University of Cape Town approved the SA-NIDS study (697/2016) and written consent was obtained from study participants. The Biomedical Research Ethics Committee at the University of KwaZulu-Natal (BE 111/14) approved the use of the SA-NIDS data.

### Statistical analysis

First, the study participants’ socio-demographic and clinical profile details at first entry/assessment into the SA-NIDS were summarized using descriptive analysis. Second, we examined their exposure to cumulative community disaster, this being the distribution and types of hazards. Lastly, we investigated the relationship between exposure to disaster and depression by fitting a generalized estimating equation (GEE) log-binomial regression model, which accounts for repeated measures (where disaster exposure, depression status, and socio-demographic characteristics can vary over time). Each regression model was further adjusted for the socio-demographic variables available, as described in the previous sub-section. Although not the primary focus of our investigation, we fitted additional regression models to investigate exposure to disaster stratified by socio-economic status, acknowledging that there are gendered [49] and socio-economic susceptibility to disasters [50], in addition to the main analysis. All analyses were adjusted by post-stratification weight to allow the results to better represent the South African population. Further details about the SA-NIDS post-stratification weight can be found elsewhere [51]. All statistical analyses were based on two-sided test, with STATA 17 being used to analyze the data.

## Results

### Socio-demographic and clinical profiles

Our sample participants consisted of 37,884 adults who participated in the SA-NIDS study at least once between wave 1 (2008) and wave 5 (2017). The participants’ socio-demographic characteristics at first entry/assessment into the SA-NIDS are described in Table 1. The majority of participants in the incident cohort were female (n = 9,647; 52.1%) and Black African (n = 13,785; 79.0%), while the overall mean age was 31.8 years (SE = 0.37) and the depression score 5.1 (SE = 0.05).

### Exposure to cumulative community disaster

The total number of study participants exposed to (i.e. reside in a community affected by) disaster hazard in the incident cohort was 2,986, with only 4.8% being exposed to a disaster hazard more than once. Broken by time periods, the number of study participants exposed to disaster hazards were 381 (5.2%, Wave 1), 697 (7.2%, Wave 2), 467 (2.0%, Wave 3), 632 (2.6%, Wave 4) and 809 (10.8%, Wave 5). The types of disaster exposure experienced by study participants were flood (n = 1,164,40.3%), drought (n = 1,075, 27.6%), mass unrest due to xenophobia (n = 381, 17.9%), agricultural loss due to fire (n = 136, 10.1%), tornado (n = 128, 2.6%), or damaged road network due to rain (n = 102, 1.7%).

### Exposure to disaster and depression

The results of the relationship between exposure to disaster and depression based on the GEE model are provided in Table 2. The adjusted regression indicates that exposure to a disaster was associated with significant depressive symptoms (adjusted relative risk [aRR] = 1.20, p<0.01, 95% CI: 1.09–1.33). The results pertaining to the role of disaster in socio-economically vulnerable groups are provided in Table 3. The regression models indicate exposure to disaster was associated with significant depressive symptoms among women [but not among men] (model 1: aRR = 1.23, p<0.01, 95% CI: 1.09–1.38), Black African [but not among other population group] (model 2: aRR = 1.21, p<0.01, 95% CI: 1.09–1.36), non-tertiary [but not among tertiary and above educational attainment group] (model 3: aRR = 1.20, p<0.01, 95% CI: 1.08–1.33), and bottom 80% income group [but not among top income quartile group] (model 4: aRR = 1.24, p<0.01, 95% CI: 1.11–1.38). We did not detect a significant role of exposure to disaster on depression among older adults ages 65+ [not reported in Table 3 due to space limitation].

## Discussion

This study investigated the relationship between exposure to community disaster and depression at a national scale in South Africa, and yielded two main findings. First, we found that exposure to a cumulative community disaster was significantly associated with first onset of depression, even after controlling for multiple socio-demographic factors. Second, we found significantly likelihood of first depression onset among females, Black African, and individuals with lower education attainment or income, due to cumulative community disaster. To the best of our knowledge, this is the first study that established an association between exposure to disaster and depression at a national scale in SSA, for both overall and socially vulnerable groups [at the time of this report]. Our findings are consistent with much of the systematic review evidence [13–17] that point to the depression challenges of individuals exposed to such stressful and catastrophic events. It is also not a surprise to find the association between cumulative community disaster exposure and first onset of depression among certain socially vulnerable populations (i.e. women, Black African, unemployed and low-income household), given the South African historical context that is marked by the legacy of patriarchal social structures [54] and persistent poverty [40].

Although our findings are consistent, that socially vulnerable populations may be at greater risk of depression challenges from disasters, we did not detect the impact on older populations, which warrants further discussion of possible hypotheses [55]. The *exposure hypothesis* contends that older individuals suffer psychological challenges due to their limited ability to relocate at short notice, and the interruption of care for their chronic health condition [55–58]. Systematic review/meta-analyses note that disaster [55, 58] did not demonstrate greater likelihood of depression in older adults. In general, according to a previous SA-NIDS study [59], depression increased with older ages, with the most dramatic increase occurring between young and middle adulthood in South Africa. Although diminished individual agency of young adults, particularly women, within the patriarchal social structure is well-established in South Africa [54], it may be the opposite for older adults (i.e. where there is the expectation of being supported by their adult children), based on the *burden hypothesis*. Although we acknowledged that families are often unable to provide filial care, as well as the diminished roles/status of older South Africans with urban progression, it is also possible, based on the *inoculation/maturation hypothesis*, that older adults’ life experiences (of disaster) may have mitigated adverse mental health outcome by developing a level of resilience to unexpected events. Older adults endured the militarized history of South African violence, which was pervasive under the Apartheid regime [60].

Interpreting our findings may be subject to several limitations, the first being that we lacked clinical diagnosis data on depression outcome. Second, children are often regarded as being vulnerable to disasters, but were not included due to the SA-NIDS not having data on depression for that population during our study period; a future study in this regard being warranted. Third, we acknowledged that a state of disaster declaration is a decision made by politicians, albeit on their advisors’ advice. Elsewhere, several studies point to political consideration, such as proximity to election [61], as contributing to disaster declaration [62, 63] and the associated decisions regarding allocation/rejection of financial assistance, given budgetary limitation [64]. Although there is no formal study, to the best of our knowledge, about the political factors that contribute to disaster declaration in South Africa, the constitutionality of section 27 of the Disaster Management Act [18] (pertaining to the national government’s authority to declare a state of disaster) is often debated. Given that disasters’ classification occurs in a political space (independent of depression status), this non-differential random misclassification may influence our study outcome (by underestimating the strength of association between exposure to disaster and depression). Furthermore, definition of disaster often varies between national and international entities. For example, an annotation on United Nations Office for Disaster Risk Reduction’s (UNDRR) definition of disaster [65] includes phrase “*may test* or exceed the capacity of a community...” which may be broader compared to the definition under the South African Disaster Management Act, 2002 (Act No. 57 of 2002) [18]. Further research on the internationally defined disasters and depression may be warranted for better cross-national comparison of results. Fourth, although our study highlights the importance of religious involvement, it is based on a crude measure. Religion is a deeply personal issue, and further qualitative, in-depth studies are needed. Lastly, residual confounding may be substantial in our observational epidemiology study. For this reason, we refrain from any causal inference about the relationship between disaster and depression.

Nevertheless, this was the first study, to our knowledge, that speaks to the danger of disasters on a mental health outcome using nationally representative household panel survey data that is unique in SSA. Regarding the long-term policy recommendation, ecosystem-based disease risk reduction (Eco-DRR) strategies need to be prioritized that involve sustainable management, conservation, and the restoration of ecosystem services that provide protection from disaster, and promote resilience agriculture [66] and health/mental health [67, 68]. In terms of potential short-term policy intervention, the experience of the economic downturn caused by the COVID pandemic in South Africa highlighted the need for a robust and inclusive social protection system [69] that can mitigate the effect of disaster, which Amartya Sen refer to as “the dangers of sudden deprivation” [70]. Given the past nationally representative evidence on the relationship between depression and food insecurity in South Africa [43], we make an argument for food security grants that not only offer protection against income loss, but also from crop loss due to disasters. SSA is regarded as the world’s most vulnerable region regarding the impact of climate change [8], where livelihoods are heavily dependent on rain-fed subsistence agricultural activities [71]. Given the limited repeated measures in the SA-NIDS, we could not incorporate food insecurity into the analysis. Nevertheless, our findings [not reported] suggest that individuals who experienced food insecurity early on in the SA-NIDS study, and are exposed to a disaster, had significantly greater depressive symptomatology.

Disasters are both negative individual and collective experiences [72], where the devastation of a community means the loss of ties, sense of belonging, and livelihoods [73]. Our study broadly reaffirms the multi-level needs for a sustainable development policy, and the implementation of a broad disaster risk reduction plan, to help both protect socially vulnerable individuals, and empower communities that support them, from the consequence of disasters. We detected the potential important role that religion played against depression among socially vulnerable populations during our analysis stage, which is often overlooked in the planetary health debate. Although we acknowledged that the perceived importance of religious activity (measure available in SA-NIDS) is crude, and does not necessarily equate to actual practice [74], the benefit derived from religious activity [73] (e.g. empathy through dialogue and human connection, mutual support for enhancing resiliency and facilitating recovery), and its institutions, may be the only source of hope for some during difficult tumultuous times. In the absence of, or when there are limited, post-disaster government recovery operations in South Africa, the role of faith-based organizations (FBOs) that are deeply embedded in communities may be the only source of institutional social security system, including moral leadership, to prevent xenophobia or other reactive protest, and spearhead inclusive community.

## Conclusion

Climate change presents perhaps the most defining sustainable development challenge for sub-Saharan Africa (SSA) in the 21st century [8, 9]. The region is one of, if not the most vulnerable [10], to the impacts of climate change [11, 12]. Its temperatures are increasing faster than the worldwide average, this variable possibly being the most important key driver of climate-related risks [75]. One major consequence of rising temperatures, coupled with precipitation anomalies, is the increasing frequency and/or intensity of natural disasters (although we prefer not to use the word ‘natural’ as mentioned previously [76]), which are projected to rise in SSA [77], including long-term trends of heat stress in South Africa [78]. Our study highlights the risk posed by cumulative community disaster (i.e. exposure to community disaster), in addition to individual-level risk factors (i.e. negative adverse event) that lead to first depression onset, particularly among socially vulnerable population groups. Although pharmacotherapy and psychotherapy for clinical depression [79] at the individual level are sometimes necessary, we believe our study supports the statement that community matters and highlights the need to foster resilient communities to mitigate the impact of a hazard that is likely to be exacerbated by climate change. In the absence of prevention, we reaffirm the forward-looking and hopeful spirit of the United Nation’s Sendai Framework for Disaster Risk Reduction 2015–2030 [80] to ‘Build Back Better’ communities that can thrive and even exceed the predisaster conditions.

## Figures and Tables

**Table 1:** Baseline socio-demographic characteristics of the study incident cohort.

		Overall	
		n	%
Gender:	Male	7,608	47.9%
	Female	9,647	52.1%
Race/ethnicity:	Black African	13,785	79.0%
	Non-Black African (Coloured‡, Asian/Indian, White)	3,470	21.0%
Age category:	15–19	5,827	31.6%
	20–24	2,069	12.3%
	25–29	1,642	10.6%
	30–34	1,382	9.7%
	35–64	5,330	30.7%
	65+	1,004	5.1%
Education:	Less than high school	1,391	5.5%
	Completed high school	12,271	66.9%
	Beyond high school	3,592	27.6%
Employment status:	Not employed	1,687	63.4%
	Employed	15,506	36.6%
Religiosity:	Not important at all/Unimportant	1,687	10.2%
	Important/Very Important	15,506	89.8%
Household income:	Lowest 20%	3,140	16.6%
	Low/Middle 20%	3,283	16.2%
	Middle 20%	3,553	17.7%
	Middle/High 20%	3,689	20.7%
	Highest 20%	3,590	28.7%
Residence:	Rural	9,151	40.6%
	Urban formal	7,032	50.8%
	Urban informal‡‡	1,044	8.6%

‡ The “coloured” is term used by Statistics South Africa [52], a South African ethnic label that may include children/ descendants from Black-White, Black-Asian, Black-Colored, and White-Asian unions [53].

‡‡ Contrary to urban formal areas, urban informal areas are settlements with limited formal urban planning [52]. % are adjusted based on post-stratification weight to better match population estimates produced by Statistics South Africa. Mean age and SE were 31.8 and 0.37 respectively. https://doi.org/10.1371/journal.pclm.0000024.t001

**Table 2:** Association between exposure to disaster and depression based on GEE model.

		Overall			
		aRR	p	95% CI	
Gender:	[Male]				
	Female	1.07	0.05	0.99	1.15
Population group:	[Non-Black African]				
	Black African	1.42	<0.01	1.26	1.61
Age category:	[15–19]				
	20–24	1.99	<0.01	1.74	2.27
	25–29	2.29	<0.01	1.99	2.63
	30–34	2.18	<0.01	1.87	2.55
	35–64	2.27	<0.01	2.02	2.56
	65+	2.53	<0.01	2.16	2.97
Highest educational attainment:	[Beyond high school]				
	High school not completed	1.24	<0.01	1.08	1.42
	Completed high school	1.15	<0.01	1.05	1.26
Employment status:	[Employed]				
	Unemployed	1.15	<0.01	1.06	1.25
Religiosity:	[Not important at all/Unimportant]				
	Important/Very Important	0.85	<0.01	0.76	0.96
Household income quintile:	[Highest income group - 5th quartile]				
	Lowest income group - 1st quartile	1.51	<0.01	1.33	1.70
	2nd quartile	1.49	<0.01	1.32	1.69
	3rd quartile	1.35	<0.01	1.19	1.54
	4th quartile	1.19	<0.01	1.05	1.36
Residential area:	[Rural]				
	Urban Formal	1.32	<0.01	1.23	1.41
	Urban Informal	1.27	<0.01	1.13	1.44
Exposure to adverse negative event:	[No]				
	Yes	1.15	<0.01	1.04	1.27
Exposed to disaster:	[No]				
	Yes	1.20	<0.01	1.09	1.33

The regression model adjusted based on post-stratification weight (from final observation of the individual panel) to reflect more recent population estimates produced by Statistics South Africa. Number of observations per participant ranged from 1 to 5 with average being 3 for the fitted GEE model above. Significance of main finding did not change when CES-D was kept as continuous outcome for the exposed to community disaster variable (adjusted β = 0.25, p<0.01). https://doi.org/10.1371/journal.pclm.0000024.t002

**Table 3:** Association between exposure to disaster and depression based on GEE model by groups at risk.

		Model 1: Sex				Model 2: Race				Model 3: Education				Model 4: Income			
		[Female only]				[Black African only]				[Non-tertiary only]				[Bottom four quartile only]			
		aRR	p	95% CI		aRR	p	95% CI		aRR	p	95% CI		aRR	p	95% CI	
Gender:	[Male]																
	Female					1.08	0.03	1.01	1.16	1.08	0.06	0.99	1.17	1.06	0.11	0.99	1.15
Population group:	[Non-Black African]																
	Black African	1.54	<0.01	1.32	1.80					1.28	<0.01	1.12	1.46	1.19	0.01	1.04	1.36
Age category:	[15–19]																
	20–24	1.96	<0.01	1.64	2.34	2.03	<0.01	1.78	2.33	2.14	<0.01	1.84	2.48	1.92	<0.01	1.67	2.22
	25–29	2.09	<0.01	1.74	2.51	2.36	<0.01	2.05	2.73	2.33	<0.01	1.97	2.75	2.19	<0.01	1.89	2.55
	30–34	2.30	<0.01	1.88	2.80	2.24	<0.01	1.91	2.63	2.31	<0.01	1.92	2.77	2.18	<0.01	1.84	2.58
	35–64	2.30	<0.01	1.97	2.69	2.34	<0.01	2.07	2.64	2.30	<0.01	2.02	2.61	2.24	<0.01	1.97	2.54
	65+	2.72	<0.01	2.23	3.33	2.71	<0.01	2.29	3.20	2.69	<0.01	2.30	3.14	2.54	<0.01	2.14	3.02
Highest educational attainment:	[Beyond high school]																
	High school not completed	1.24	0.01	1.05	1.46	1.17	0.02	1.02	1.34					1.17	0.03	1.01	1.36
	Completed high school	1.18	<0.01	1.05	1.32	1.10	0.03	1.01	1.21					1.09	0.10	0.98	1.20
Employment status:	[Employed]																
	Unemployed	1.06	0.33	0.95	1.18	1.18	<0.01	1.08	1.28	1.15	<0.01	1.04	1.28	1.17	<0.01	1.07	1.28
Religiosity:	[Not important at all/ Unimportant]																
	Important/Very Important	0.84	0.05	0.70	1.00	0.86	0.02	0.77	0.97	0.87	0.04	0.77	0.99	0.85	<0.01	0.75	0.96
Household income quintile:	[Highest income group - 5th quartile]																
	Lowest income group - 1st quartile	1.50	<0.01	1.27	1.75	1.37	<0.01	1.21	1.56	1.37	<0.01	1.19	1.59				
	2nd quartile	1.41	<0.01	1.20	1.66	1.34	<0.01	1.18	1.53	1.39	<0.01	1.20	1.60				
	3rd quartile	1.36	<0.01	1.14	1.62	1.17	0.02	1.03	1.34	1.20	0.03	1.02	1.40				
	4th quartile	1.21	0.02	1.03	1.42	1.00	0.97	0.88	1.15	1.06	0.47	0.91	1.23				
Residential area:	[Rural]																
	Urban Formal	1.31	<0.01	1.19	1.43	1.31	<0.01	1.21	1.41	1.32	<0.01	1.22	1.44	1.25	<0.01	1.16	1.35
	Urban Informal	1.26	<0.01	1.09	1.47	1.29	<0.01	1.14	1.45	1.26	<0.01	1.09	1.45	1.25	<0.01	1.09	1.42
Exposure to adverse negative event:	[No]																
	Yes	1.07	0.32	0.94	1.22	1.13	0.01	1.03	1.24	1.18	<0.01	1.05	1.33	1.12	0.05	0.99	1.25
Exposed to disaster:	[No]																
	Yes	1.23	<0.01	1.09	1.38	1.21	<0.01	1.09	1.36	1.20	<0.01	1.08	1.33	1.24	<0.01	1.11	1.38

https://doi.org/10.1371/journal.pclm.0000024.t003

## Data Availability

All data are in a public data repository without restriction from https://www.datafirst.uct.ac.za/dataportal/index.php/catalog/712 and https://opengazettes.org.za/.
